# Lower Body Acceleration and Muscular Responses to Rotational and Vertical Whole-Body Vibration at Different Frequencies and Amplitudes

**DOI:** 10.1177/1559325818819946

**Published:** 2019-01-06

**Authors:** Lisa N. Zaidell, Ross D. Pollock, Darren C. James, Joanna L. Bowtell, Di J. Newham, David P. Sumners, Katya N. Mileva

**Affiliations:** 1 Sport and Exercise Science Research Centre, London South Bank University, London, United Kingdom; 2 Centre of Human and Applied Physiological Sciences, King’s College London, London, United Kingdom; 3 Sport and Health Sciences, University of Exeter, Exeter, United Kingdom; 4 Inside Technology, Darlington, United Kingdom

**Keywords:** whole-body vibration, acceleration, electromyography, transmission, spine

## Abstract

**Aim:**

The aim of this study was to characterize acceleration transmission and neuromuscular responses to rotational vibration (RV) and vertical vibration (VV) at different frequencies and amplitudes.

**Methods:**

Twelve healthy males completed 2 experimental trials (RV vs VV) during which vibration was delivered during either squatting (30°; RV vs VV) or standing (RV only) with 20, 25, and 30 Hz, at 1.5 and 3.0 mm peak-to-peak amplitude. Vibration-induced accelerations were assessed with triaxial accelerometers mounted on the platform and bony landmarks at ankle, knee, and lumbar spine.

**Results:**

At all frequency/amplitude combinations, accelerations at the ankle were greater during RV (all *P* < .03) with the greatest difference observed at 30 Hz, 1.5 mm. Transmission of RV was also influenced by body posture (standing vs squatting, *P* < .03). Irrespective of vibration type, vibration transmission to all skeletal sites was generally greater at higher amplitudes but not at higher frequencies, especially above the ankle joint. Acceleration at the lumbar spine increased with greater vibration amplitude but not frequency and was highest with RV during standing.

**Conclusions/Implications:**

The transmission of vibration during whole-body vibration (WBV) is dependent on intensity and direction of vibration as well as body posture. For targeted mechanical loading at the lumbar spine, RV of higher amplitude and lower frequency vibration while standing is recommended. These results will assist with the prescription of WBV to achieve desired levels of mechanical loading at specific sites in the human body.

## Introduction

Degenerative losses in both skeletal muscle and bone mass present a major challenge to health for the aging population. Therefore, interventions to maintain and improve musculoskeletal strength in at-risk populations are essential. Whole-body vibration (WBV) can provide mechanical loading to the body,^1^ and in some cases, it is thought to be associated with increased muscle activation.^2,3^ Correspondingly, since loading and muscle activation are important for bone remodeling,^4^ WBV has been used as a novel countermeasure for sarcopenia^5^ and osteoporosis,^6^ which may help reduce the incidence of bone fractures. Although WBV can be beneficial for maintaining or increasing bone and muscle strength in younger and older populations, this is not always the case^5,7,8^ and differing results may be related to habitual activity/loading. Indeed, there is variability in response to WBV as changes in bone structure after WBV are not observed across all skeletal sites^9^ and WBV-induced muscular activation varies between muscles.^3,10^

**Figure 1.**
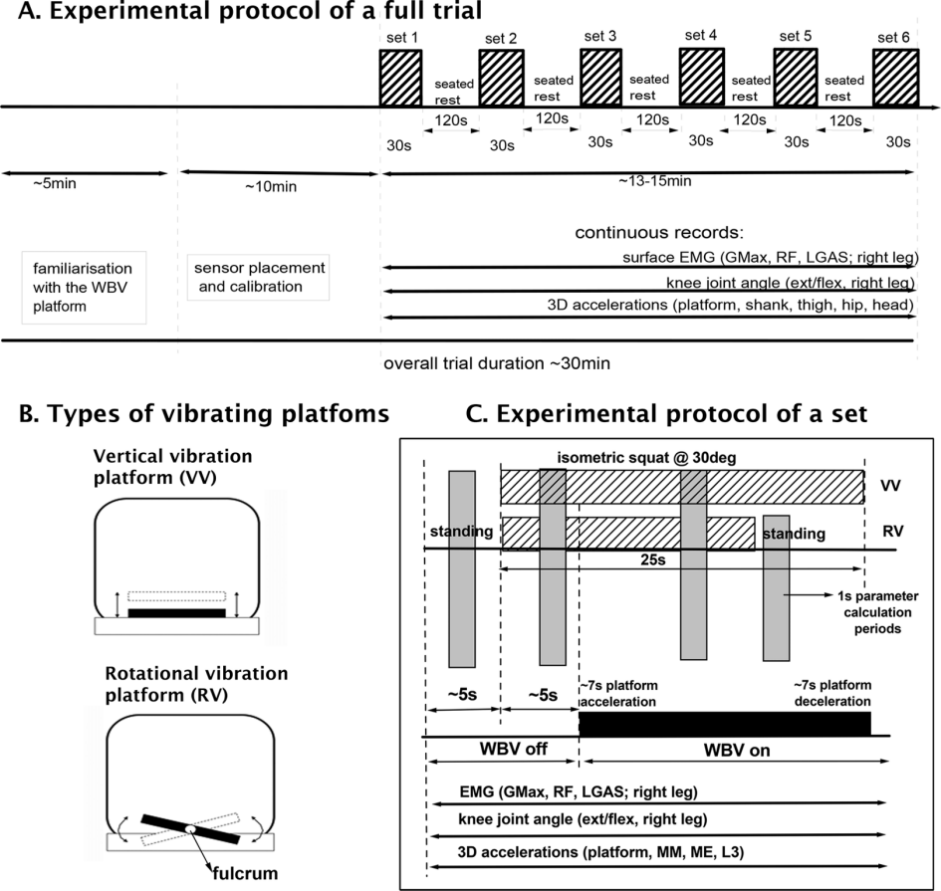
Schematic diagram illustrating (A) the experimental protocol of a full trial, (B) the oscillation direction across the fulcrum of the vertical vibration (VV) and the rotational vibration (RV) platforms, and (C) the procedures during a single experimental set. During each set, the vibration was delivered at different randomized combination of vibration frequencies (20, 25, and 30 Hz) and amplitudes (1.5 and 3 mm).

Disparities in the physiological responses to WBV may in part be due to differences in the responsiveness and sensitivity of tissues within the body to particular vibration signals. Furthermore, the response to WBV may be reliant on vibration transmission through the body, which in turn is dependent on vibration intensity (frequency and amplitude^11-13^), direction,^14,15^ and posture.^11,14^ In a practical setup, what the user can achieve through the control panel of the WBV device could also influence the physiological outcomes. Across studies, amplitudes of <1 to 10 mm peak-to-peak and frequencies between 5 and 50 Hz are generally used, which in combination have the potential to impose short-duration gravitational loads up to 50 g. In addition, the direction of vibration can be delivered by vertical or rotational oscillating platforms (Figure 1B). With vertical vibration (VV), erect standing cannot be tolerated due to high vibration transmission to the head,^16^ while with rotational vibration (RV), standing is suggested. Hence, postural differences add to an already complex paradigm for optimal WBV dose prescription.

There has been little distinction made in the literature between the physiological effects evoked by RV and VV at differing frequencies and amplitudes or whether the vibration output of platforms is consistent with the defined input settings. To achieve desired outcomes from WBV interventions, consideration should be given to the vibration intensity and direction and how they influence transmission throughout the body. Therefore, the aim of this study was to characterize the platform acceleration and the acceleration and neuromuscular response at various sites in the lower body to RV and VV at different frequencies and amplitudes. Furthermore, the effect of posture (squatting vs standing) during RV on these measures was also assessed.

## Methods

### Participants

Twelve healthy males (aged 32 [2] years, mass 81 [4] kg, height 1.77 [0.02] m; mean [standard deviation, SD]) participated in this study. Individuals who had any musculoskeletal disorders, recent fractures, and cardiovascular or neurological conditions were excluded. The University Research Ethics Committee approved the study protocol, and written informed consent was obtained from each participant.

### Experimental Conditions

On 2 occasions separated by at least 7 days, each participant performed identical protocols on either an RV (Galileo 2000; Novotec Medical GmbH, Pforzheim, Germany) or a VV (Fitvibe Medical; GymnaUniphy, Belgium) platform, which consisted of 6 sets (20-L; 20-H; 25-L; 25-H; 30-L; 30-H) of WBV with different combinations of vibration frequency (20, 25, and 30 Hz) at low (L: 1.5 mm peak-to-peak) and high (H: 3.0 mm peak-to-peak) amplitude (Figure 1A). Each set started with two 5-second periods of nonvibrated standing and squatting (WBV off) followed by a 20-second WBV squatting exercise (WBV on). During RV, after 15 seconds of WBV squat, participants were required to stand straight with locked knees for 5 seconds (Figure 1C). The order of sets was randomly allocated on each occasion. Two minutes of seated rest separated each set.

During each set, participants assumed a static squat posture (30° external knee flexion) with arms crossed and held at the chest while looking straight ahead. The angle of squat was controlled by the participant using visual feedback displayed from a knee electrogoniometer. For RV, amplitude is controlled by varying the distance of the feet from the fulcrum of the platform. The foot separation required during RV to achieve the 2 vibration amplitudes was replicated on the VV platform in order to fully match the body posture across trials. All testing was performed with the participants wearing socks and without shoes.

### Data Acquisition

#### Electromyography

Muscle activity of the *m*. gastrocnemius lateralis (LGas), *m*. rectus femoris (RF), and *m.* gluteus maximus (GMax) from the right leg was recorded using an 8-channel Bagnoli desktop electromyography (EMG) system with DE-2.1 single differential electrodes (99.9% Ag, 10 mm length, 1 mm width, 10 mm pole spacing, common mode rejection ratio >80 dB; Delsys Inc, Boston, Massachusetts). The EMG signals were amplified (×1000), band pass filtered between 20 and 450 Hz, and transferred online to a computer via A-D conversion (CED 1401; Cambridge Electronic Design Limited, Cambridge, United Kingdom) with a sampling frequency of 2000 Hz. The EMG electrodes were positioned over the muscle belly in accordance with SENIAM guidelines.^17^ Electrodes were orientated parallel to the longitudinal axis of the muscle fiber and secured with double-sided adhesive tape after the site was shaved, lightly abraded, and cleaned with alcohol wipes. The reference electrode was placed over the patella, and all cables were twisted together and taped to the body to reduce electrical and mechanical interference.

#### Accelerometry

Triaxial accelerations (anterior–posterior, AP; medial–lateral, ML; and vertical, Ve) were recorded using light-weight sensors (ACL300 [±10 g range], DataLOG; Biometrics Ltd, United Kingdom) that were calibrated to a global axis before being attached to the loaded platform (Plat), the distal anteromedial aspect of the tibia—medial malleolus (MM), medial epicondyle of the femur (ME), and lumbar vertebra 3 (L3). The data were sampled at 1000 Hz and digitized via an A-D converter (CED1401 power; Cambridge Electronic Design Limited). A custom written program (Spike 2; Cambridge Electronic Design Limited) was used to trigger and synchronously record EMG and acceleration signals. All data were stored for offline analysis.

#### *Knee joint angl*e

The angular displacement profile of the knee joint (flexion/extension) was continuously recorded via a preamplified biaxial electrogoniometer (SG150; Biometrics System, United Kingdom) centered over the lateral epicondyle of the femur with one end plate attached to the shank and aligned to the lateral malleolus of fibula and the other to the thigh and aligned to the greater trochanter of the femur using double-sided medical tape. The knee flexion angle was set to 0 during neutral standing position.

### Data Analysis

The files containing synchronized EMG, knee joint angle, and acceleration data were analyzed in Spike 2 software (Cambridge Electronic Design Limited) using custom written scripts. Records representing 1 second of data collected during squatting (RV and VV) and standing (RV only) from each set were chosen for analysis. Furthermore, 1-second baseline data (no vibration), recorded at the beginning of each condition, were analyzed and used for normalization. The DC offset was removed from the acceleration and EMG signals to account for gravitational acceleration and movement artifact, respectively. The root mean square (RMS) amplitude was then derived from the 1-second EMG (µV) and acceleration (g) profiles. Vibration-induced artifacts in the raw EMG signals were attenuated using a spectral smoothing procedure.^18^ Absolute RMS EMG amplitude recorded during squatting in RV and VV (and standing in RV) is presented for all muscles. Muscle activity during WBV while squatting was normalized to baseline (nonvibrated squatting) to account for the posture-induced muscle activity. However, during nonvibrated standing, muscle activity was within 2SD of background EMG baseline level; thus, only absolute data were compared between the sets.

The 3 axes of acceleration were considered individually for each platform but also used to calculate resultant (RES) acceleration (Equation 1). For skeletal sites: MM, ME, and L3, accelerometers were positioned to correspond to AP, ML, and Ve directions. However, the curvature of the landmarks resulted in slightly different orientations of accelerometers across participants, and thus, the individual planes of movement were not identical across participants. To overcome this, RES was determined and analyzed to represent the total magnitude of mechanical loading.

1 $$\text{RES}=\sqrt{{\text{AP}}^{\text{2}}+{\text{ML}}^{\text{2}}+{\text{Ve}}^{\text{2}}}$$

### Statistical Analyses

Data were summarized as mean (SD). Acceleration and EMG data were not normally distributed (Shapiro-Wilk); therefore Friedman test for repeated measures (SPSS 18.0) was used to compare EMG and acceleration: (1) between RV vs VV squatting at corresponding frequency and amplitude, (2) between vibration frequencies (0, 20, 25, and 30 Hz) for each vibration direction, (3) between vibration amplitudes (low and high) for each vibration direction, and (4) between standing and squatting (RV only). Significance was set at *P* < .05 in all cases.

## Results

### Acceleration Amplitude of the VV and RV Platforms

**Figure 2.**
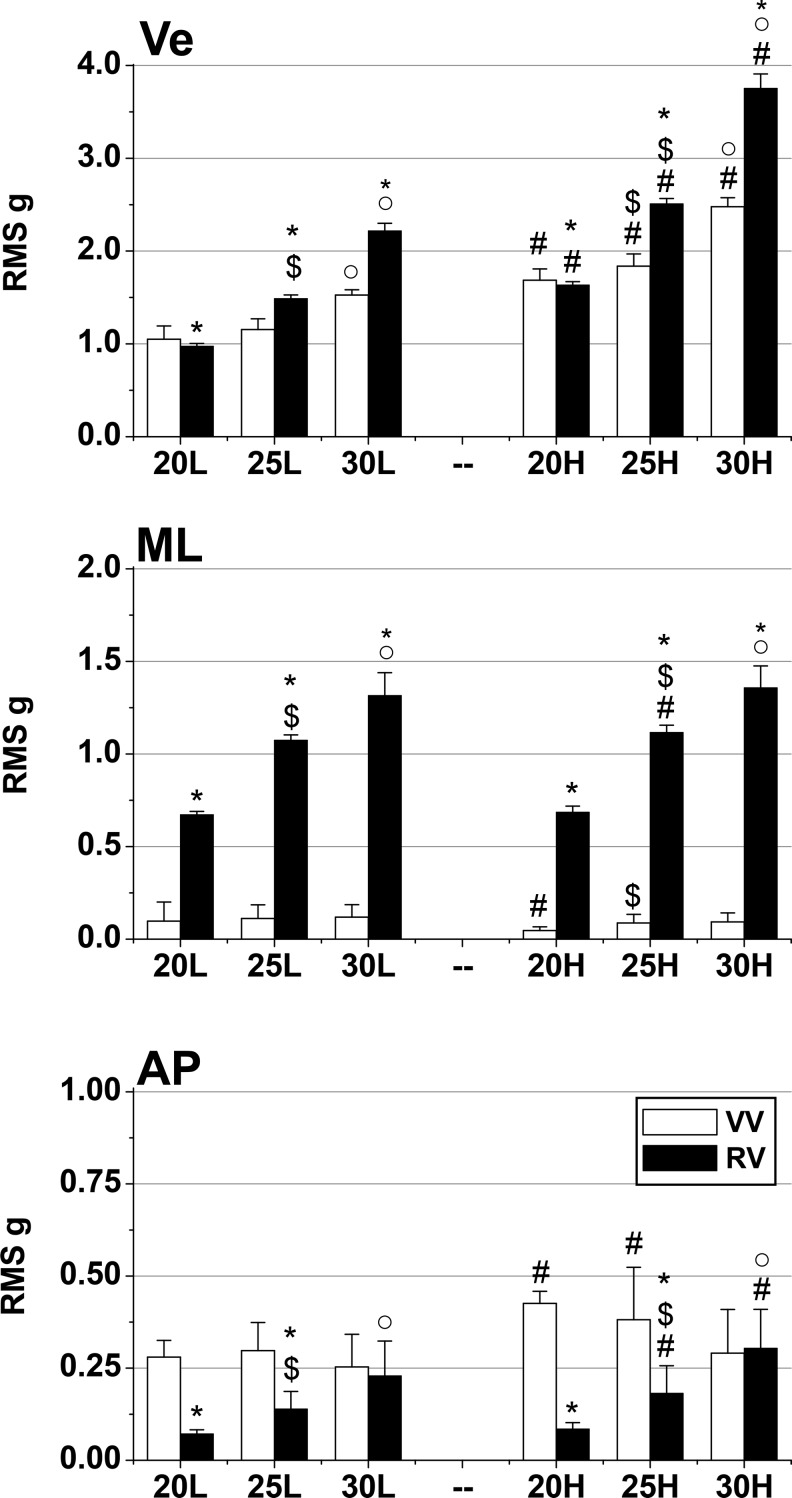
Triaxial accelerations produced by the vertical and rotational whole-body vibration (WBV) platforms during different combinations of vibration frequency and amplitudes. Mean (SD) platform acceleration (root mean square [RMS] g) in vertical (Ve), mediolateral (ML), anterior–posterior (AP) directions. Significantly different (*P* < .05): *versus vertical vibration (VV), ^#^versus low amplitude vibration, ^$^versus 20 Hz, ˚versus 25 Hz.

**Figure 3.**
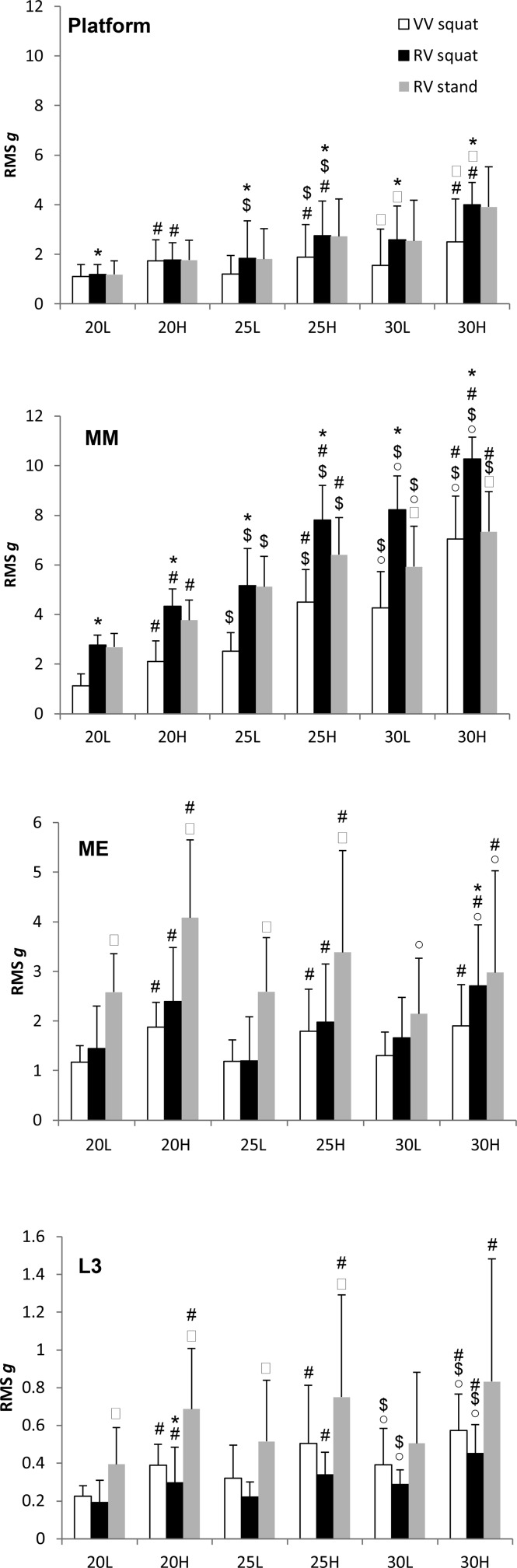
Effect of oscillation direction, frequency, and amplitude of vibration on the resultant acceleration (mean[SD]) recorded during squatting and standing on a vertical and rotational platforms at different sites: platform surface, medial malleolus (MM), medial epicondyle (ME), lumbar spinal vertebral process 3 (L3), and combination of vibration frequencies (20, 25, and 30 Hz) and amplitudes (L: low; H: high). Significantly different (*P* < .05): *versus vertical vibration (VV); ^#^versus low amplitude vibration; ^$^versus 20 Hz; ˚versus 25 Hz; ^□^versus squat posture.

Despite setting the WBV platforms to produce the same vibration frequencies and amplitudes, the recorded acceleration output significantly differed between RV and VV conditions along all 3 axes (Figure 2). The differences between platforms were frequency dependent; vertical acceleration (Ve) was greater at 20 Hz with VV (L, H: *P* < .03) and at 25 and 30 Hz with RV (*P* =.001). Mediolateral acceleration was greater with RV (*P* = .001), and AP acceleration was greater at 20 and 25 Hz with VV (L, H: *P* ≤ .004). Greater RES occurred with RV (*P* < .004) except at 20-H (Figure 3).

Higher vibration frequencies resulted in greater Ve acceleration for both platforms (*P* = .001) with greater ML (*P* = .001) and AP (*P* < .03) accelerations for RV only. The RES acceleration was also greater at higher frequencies (*P* ≤ .004) except VV 25-L versus 20-L. High-amplitude vibration (3 vs 1.5 mm) led to greater Ve (*P* = .001), ML (RV: 25 Hz, *P* = .001; VV: 20 Hz, *P* = .004), AP (VV: 20-H, 25-H; RV: 25-H, 30-H; *P* < .03; Figure 2), and RES (*P* = .001; Figure 3).

### Resultant Acceleration Amplitude at Skeletal Sites During Squat Posture with VV and RV

#### Medial malleolus

At all frequency and amplitude combinations, greater acceleration at the MM occurred with RV than VV (*P* < .03; Figure 3). Medial malleolus acceleration was greater at higher frequencies (*P* < .004) and amplitude (*P* < .004).

#### Medial epicondyle

Rotational vibration and VV produced similar acceleration at ME (Figure 3), except at 30-H where acceleration was greater with RV (*P* = .004). Similar ME acceleration was observed across frequencies, except RV 25-H was greater than 30-H (*P* = .001) and greater RES occurred with higher amplitude WBV (*P* < .004).

#### L3 vertebral spinal process

Acceleration tended to be higher for RV than VV but reached statistical significance only at 20-H (*P* = .021; Figure 3). Higher frequencies of vibration resulted in greater RES, but this difference was significant only between 25 and 30 Hz (*P* < .03). Greater RES occurred at higher amplitude WBV (*P* ≤ .004).

### Effect of Posture on Resultant Acceleration During Rotational Vibration

#### Medial malleolus

The RES was similar during standing and squatting at 20 and 25 Hz; greater RES occurred with squatting than standing at 30 Hz (30-L: *P* = .001; 30-H *P* = .004; Figure 3). Increasing the amplitude (*P* = .001) and frequency (*P* < .03) of vibration led to greater acceleration at MM during both standing and squatting.

#### Medial epicondyle

The RES was greater during standing versus squatting at 20 and 25 Hz (*P* ≤ .03; Figure 3). During standing, acceleration increased with frequency only for 25 versus 30 Hz (*P* < .03). Acceleration during standing was greater at high-amplitude vibration (*P* ≤ .004).

#### L3 vertebral spinal process

The RES was greater with standing versus squatting at 20 and 25 Hz (*P* ≤ .004; Figure 3). Acceleration during standing increased with greater vibration amplitude (*P* < .03), but not frequency.

### Electromyography RMS Amplitude

#### Lateral gastrocnemius

**Figure 4.**
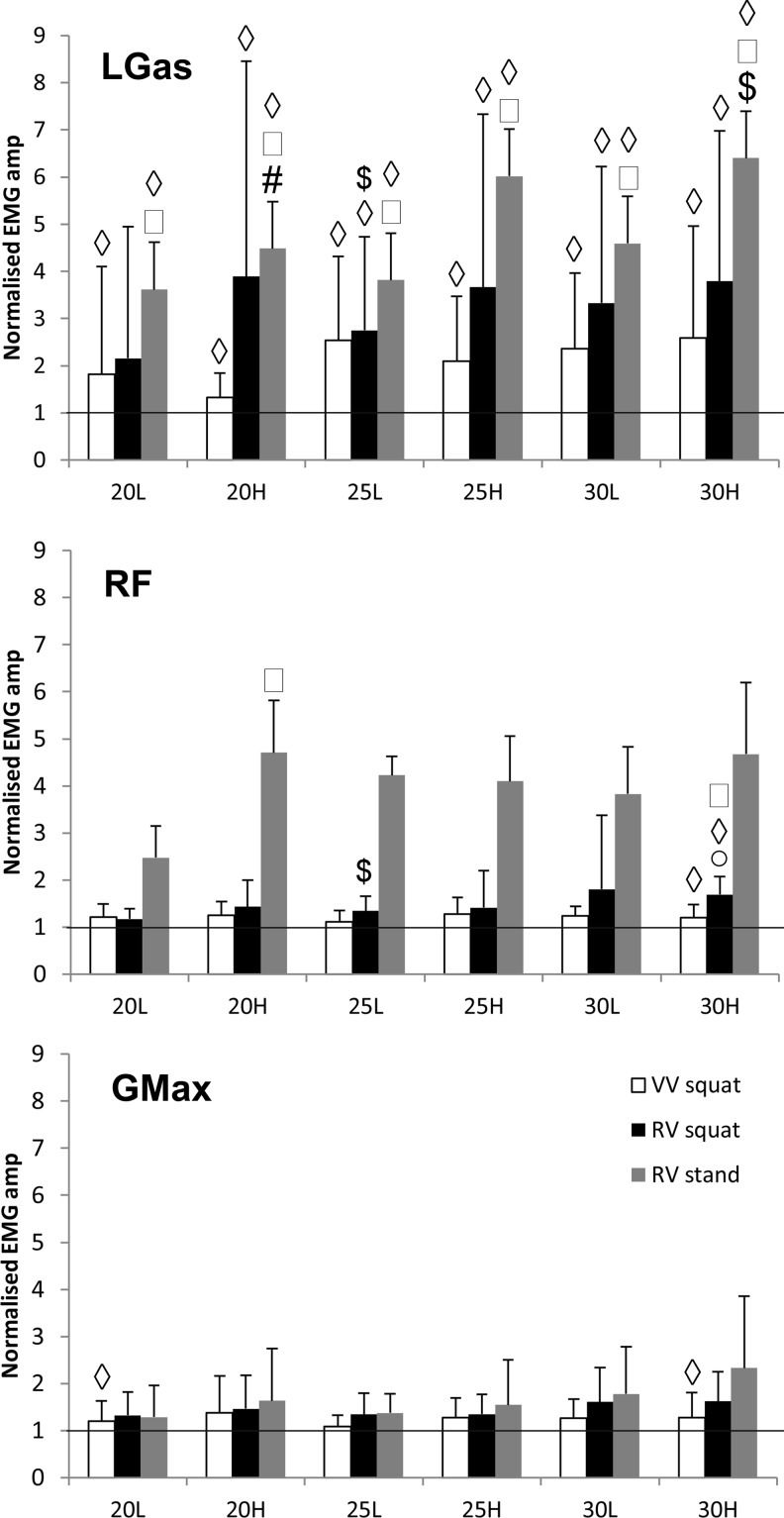
Effect of oscillation direction, frequency, and amplitude of vibration, and body posture on muscle activation during squatting or standing on a vertical or rotational whole-body vibration platform operating at combinations of vibration frequencies (20, 25, and 30 Hz) and amplitudes (L: low; H: high). EMG RMS amplitude (mean [SD], n = 12) was normalized to baseline activity without vibration and recorded from: (A) m. gastrocnemius lateralis (LGas), (B) m. rectus femoris (RF), (C) m. gluteus maximus (GMax). Significantly different (*P* < .05): *versus vertical vibration (VV); ^#^versus low amplitude vibration; ^$^versus 20 Hz; ˚versus 25 Hz; ^◊^versus control (no vibration); ^□^versus squat posture.

Whole-body vibration increased the activity during squatting (VV: *P* < .04; RV: *P* < .03, Figure 4) except during RV 20-L (*P* = .25); differences in amplitude between VV and RV were not observed. During RV standing, activity increased with all WBV conditions (*P* = .001) and was greater compared with that observed during squatting (*P* < .03). Activity did not increase with vibration amplitude or frequency, except for RV 20-L versus 25-L during squatting (*P* = .021).

#### Rectus femoris

Activity increased during squatting only with vibration at 30-H (VV: *P* = .034; RV: *P* = .004; Figure 4) which was significantly greater with RV than VV (*P* = .021). During squatting, increasing the frequency of vibration increased activity only with RV (*P* ≤ .03) with no amplitude effect observed for either VV or RV. Activity was greater during standing versus squatting with 20-H RV only (*P* ≤ .03). During standing, activity increased with RV of 25-H only, and increasing the frequency and amplitude did not alter RF activity during standing RV.

#### Gluteus maximus

Activation increased significantly (vs non-vibration) during squatting with VV only (20-L, 30-H: *P* < .04; Figure 3) and was not different between RV and VV. Activity did not increase with frequency or high-amplitude vibration and was not affected by posture.

## Discussion

### Vibration Acceleration

The present investigation highlights the importance of ensuring that evaluation of WBV effects and its prescription is specific to the mode of vibration used. Despite the same input vibration characteristics (frequency and amplitude) being used, the resultant acceleration produced during RV was greater than during VV indicating that the output of the platforms does not necessarily reflect the platform settings. Although the greatest levels of acceleration were produced in the vertical (Ve) direction by both platforms, these were different between platforms. Lower Ve acceleration observed during VV at higher frequencies is likely to be explained by decreased amplitude of the VV platform with increasing vibration frequency (data not shown). This suggests an inability of the VV platform to reach the desired amplitude at higher frequencies—a finding previously reported with other VV platforms^14,19^ and recently with RV.^20^ The greater magnitudes of resultant acceleration during RV compared with VV demonstrate that RV will deliver higher levels of mechanical loading compared with VV.

The intensity of vibration-induced acceleration at bony landmarks on the tibia, femur, and spine was measured with the findings supporting an attenuation of vibration as it ascends proximally through the lower extremities^12,21^ due to passive^22,23^ and active^24^ damping mechanisms. At the lumbar region (L3), accelerations were reduced (up to ∼10 times) relative to those produced at platform level for both RV and VV, falling <1 RMS g and in some cases below those known to be anabolic to bone (0.3 g^25^), for example, setting of both RV and VV to 20 Hz frequency and 1.5 mm amplitude (Figure 3). Since the lumbar spine is a common site of osteoporosis,^26^ this finding is important for the use of WBV in the targeted treatment and prevention of metabolic bone disorders. Although vibration damping by leg musculature may be responsible for the negligible effects of WBV on whole-body bone mineral density (BMD), increases in lumbar spine BMD with WBV have been observed and are reported to be dependent on body posture and the direction and intensity of vibration.^27^

Greater vibration damping was shown to occur with RV across all conditions tested. Accelerations reaching L3 were generally lower with RV perhaps due to damping induced from the side-to-side motion at the hip joint.^14,28^ An important aspect of RV is its greater tolerance during standing posture which, in the current study, induced nearly 2-fold greater acceleration at L3 compared to squatting, although this was still heavily damped. This effect of posture has also been reported with VV.^29^ Notably, the greater acceleration at the lumbar spine during RV standing compared with squatting also exceeded the magnitudes produced with VV during squatting. Training studies show greater improvements in spine BMD^27,30^ with RV rather than VV, especially during standing.^27,31^ This finding is particularly pertinent for effective treatment of the lumbar region.

Reduced acceleration was observed at the medial epicondyle compared to that at the medial malleolus and demonstrates an attenuation of vibration transmission at sites more distal to the platform as previously reported.^11,13,2,32^ The knee joint may act as a major contributor to damping and better acceleration transmission is likely to occur during standing during WBV due to the “cushioning” effect of the knee flexion which modulates impact transmission during gait.^23^ Above the knee, vibration acceleration loading of ∼1 to 2.5 RMS g was observed, and thus, complete attenuation of vibration did not occur. Borer (2005)^33^ suggested that a strain threshold must be exceeded for bone remodeling; however, this may be dependent on several factors including strain direction, magnitude and rate, the number of loading cycles, and the distribution of loading.^34^ Short bouts of physical activity at intensities ≥1 g (eg, running) and ≥0.75 g (eg, slow jogging) in pre- and postmenopausal women are positively associated with bone health.^35^ Higher magnitudes of mechanical loading that occur during intense physical activity are osteogenic^36^ and may only require brief bouts or few cycles of loading.^25^ Despite reduced transmission above the knee, the level of mechanical loading is likely to represent a stimulus sufficient for bone anabolism.^37-39^ With the higher magnitudes of loading observed in the current study, depending on the targeted site, WBV exposure of short duration may elicit positive osteogenic effects.

Despite attenuation of acceleration at both knee and spine, attenuation through the body is not linear. Compared to platform levels, amplification of the resultant accelerations at the ankle was observed with both RV and VV (Figure 3), which is in line with recent research on VV^29,40^ and RV.^41^ Previous research indicate that shank acceleration is greater at lower frequencies,^11,42^ with Crewther et al^1^ reporting greater transmission during 20 Hz VV compared with 10 and 30 Hz. Friesenbichler et al^41^ reported that peak acceleration at the shank increased with increasing vibration (RV) frequency, although a concomitant decrease in vibration transmissibility was also observed from platform to shank. Here, greater acceleration at the ankle occurred at higher frequencies (and amplitude) of both RV and VV. Differences between study findings most likely relate to factors such as accelerometer placement, transmission calculation, and the vibration frequencies and amplitudes employed.

When matched for frequency and amplitude, RV imparted greater mechanical load at the ankle than VV. This is likely due to the higher magnitude of acceleration generated by the platform but may also be influenced by the direction of acceleration and differences in damping strategies employed by the musculoskeletal system.^43^ At 20 Hz high-amplitude vibration, platform acceleration was similar between RV and VV but different at the ankle. This demonstrates that the direction of vibration application alters its transmission through the foot–ankle complex. Since vibration transmission is closely related to the dynamic characteristics of the foot and ankle complex, it is possible that RV and VV impose different levels of mass loading at the foot which alters compression, stiffness, resonance frequency,^44^ and hence transmission to the shank. Standing during RV resulted in lower levels of ankle acceleration (vs RV squatting); however, these were still relatively high (up to ∼7 RMS g). Therefore, this finding requires consideration in the use of WBV with osteopenic/osteoporotic individuals. Although no adverse effects of WBV at frequencies and amplitudes similar to those used here have been reported by training studies in older populations,^39,45,46^ caution is warranted over high magnitudes of loading particularly for the fragile skeleton.^25^

### Muscle Activity

In the current study, vibration activated musculature in the shank, thigh, and hip regions in some, but not all, conditions. The *m*. lateral gastrocnemius (LGas) appeared to be most consistently activated by both RV and VV across conditions, while the *m*. rectus femoris (RF) was significantly greater than baseline only at 30 Hz high-amplitude vibration. Vibration-induced activation of the *m*. gluteus maximum (GMax) above quiet standing was seen only with VV at 20 Hz low- and 30 Hz high-amplitude vibration. GMax activity was similar in VV and RV and also during RV standing and squatting. Increases in muscular activity with WBV is not a universal finding^47^ and appears to be dependent on vibration frequency and external loading.^2^ High interindividual variability in muscle activity across a range of frequencies (30-50 Hz) has been reported.^29^ Although other research^3,48^ report that muscle activity tends to be greater with RV, the findings of the current study generally do not support this notion.^3,48^

The more consistent activation of the LGas with WBV is likely related to high vibration transmission from platform to ankle irrespective of the frequency and amplitude of vibration. However, when matched frequency and amplitude of vibration were set using the platform interface, LGas activation was similar between RV and VV despite differences in ankle acceleration. Thus, vibration transmission may not be the primary mediator of muscle activation. The similar levels of LGas activation in response to WBV at around 25 to 35 Hz may potentially be due to this frequency range being close to the muscle’s resonance frequency.^32^ The function of this muscle in postural control may also contribute to its activation during unstable standing.^49^ Indeed, greater LGas activity was observed with standing than squatting (RV), and therefore, factors other than vibration transmission,^14^ such as posture and the associated changes in joint and muscle stiffness and muscle tension, may modulate vibration-induced muscle activation.^50^

Whether vibration transmission is the modulating factor or not, it is thought that muscles more distal to the platform are less consistently activated with WBV than those more proximal.^2,32^ At thigh, greater neuromuscular activation has been shown to occur with RV^3^ and at higher frequencies and amplitudes.^2^ Activation of the *m.* vastus lateralis is more commonly reported and has been observed with both VV and RV.^3^ Activation of the *m*. vastus lataralis and medialis but not the rectus femoris during WBV of similar frequencies^34^ suggests biarticular and monoarticular muscles may produce different responses to WBV.^51^ Here, RF muscle activation was augmented by standing posture (vs squatting RV), but this only reached statistical significance with 20 Hz amplitude vibration. Consistent with the literature, activation of the GMax was lower than other lower limb muscles during WBV^13^ with no clear dose–response relationship seen with peak platform acceleration.^29^ Activation of upper leg musculature may require higher vibration amplitudes and frequencies (>4 mm and >30 Hz),^2^ while different body postures such as deep squat^48^ or dynamic exercise^52^ may be more effective. Static standing during WBV, however, has led to more pronounced muscle activation in older adults.^53^ Furthermore, inconsistent muscle activation in the current study may also be due to the brief WBV exposure period applied; longer exposures may be necessary for eliciting or maximizing the tonic vibration reflex.^42^

### Recommendations

Differences were observed between the 2 platforms through characterizing acceleration and muscular responses at various sites in the lower body to different frequencies and amplitudes and with reference to the posture assumed. These differences should be considered when designing WBV protocols. For example, the posture assumed alters the transmission of vibration through the body; if erect standing is the most practical posture to assume, then RV may be the safest platform to use to minimize head vibration. If the platform has limited frequency and amplitude settings, then different postures may be used to manipulate vibration transmission to specific body sites.

To maximize mechanical loading below the knee, the use of 30 Hz in combination with the higher amplitude seems most advantageous, particularly during squatting with RV for the parameters investigated here. However, for those with the more frail skeletons, using VV rather than RV can reduce mechanical loading at the ankle. Alternatively, adopting a standing posture during higher frequency RV or lowering the frequency and amplitude of vibration reduces loading of the lower leg.

For targeted mechanical loading of the lumbar spine, it appears optimal to adopt a standing stance on RV platform; the greatest magnitudes of acceleration were observed at high amplitude irrespective of vibration frequency. This increased transmission to the spine at 20 and 25 Hz compared with a squat posture, without affecting loading at the ankle. Given the above considerations, when targeting the lumbar spine, rotational-based WBV of 20 or 25 Hz (3.0 mm peak-to-peak amplitude) while standing, a posture more user-friendly, especially for those with balance problems, is recommended. The use of 25 Hz, 3.0 mm RV during standing also has the advantage of activating the thigh (RF) and shank (LGas) musculature, which may be beneficial for bone perfusion and muscle strengthening.

## Conclusions and Implications

- Informed choice of WBV platform and protocol should be made to achieve specific outcomes from vibration training since differences in acceleration output, transmissibility, and muscle activation exist between RV and VV of varying frequencies and ampitudes.
- Adopting a standing posture on a RV platform operating at high amplitude and lower frequencies is optimal for targeted mechanical loading of the lumbar spine and activation of the shank and thigh musculature without additional loading of the ankle joint.
- By enabling standing postures, RV may be more suitable for populations unable to maintain balance during squatting.
